# Discovery of a novel 4-pyridyl SLC-0111 analog targeting tumor-associated carbonic anhydrase isoform IX through tail-based design approach with potent anticancer activity

**DOI:** 10.3389/fchem.2025.1571646

**Published:** 2025-04-04

**Authors:** Hamada Hashem, Shadwa Abdelfattah, Hesham M. Hassan, Ahmed Al-Emam, Mohammed Alqarni, Ghallab Alotaibi, Ibrahim Taha Radwan, Kirandeep Kaur, Devendra Pratap Rao, Stefan Bräse, Abdullah Alkhammash

**Affiliations:** ^1^ Pharmaceutical Chemistry Department, Faculty of Pharmacy, Sohag University, Sohag, Egypt; ^2^ Department of Pharmaceutics and Industrial Pharmacy, Faculty of Pharmacy, Merit University (MUE), Sohag, Egypt; ^3^ Department of Pathology, College of Medicine, King Khalid University, Asir, Saudi Arabia; ^4^ Department of Pharmaceutical chemistry, College of Pharmacy, Taif University, Taif, Saudi Arabia; ^5^ Department of Pharmacology, College of Pharmacy, Shaqra University, Shaqra, Saudi Arabia; ^6^ Supplementary General Sciences Department, Faculty of Oral and Dental Medicine, Future University in Egypt, Cairo, Egypt; ^7^ Department of Chemistry, Maharaja Ranjit Singh Punjab Technical University, Bathinda, Punjab, India; ^8^ Coordination Chemistry Laboratory, Department of Chemistry, Dayanand Anglo-Vedic (PG) College, Kanpur, Uttar Pradesh, India; ^9^ Institute of Biological and Chemical Systems, Functional Molecular Systems (IBCS-FMS), Karlsruhe Institute of Technology (KIT), Karlsruhe, Germany

**Keywords:** sulphonamide, SLC-0111, carbonic anhydrase, cytotoxicity, apoptosis

## Abstract

**Introduction:** Carbonic anhydrase IX (CA IX) is a tumor-associated enzyme involved in cancer progression and survival. Targeting CA IX with selective inhibitors like SLC-0111 has shown therapeutic potential. This study aimed to develop a novel 4-pyridyl analog (**Pyr**) of SLC-0111 with enhanced anticancer activity.

**Methods:** **Pyr** was synthesized using a tail-based design and characterized by NMR. Its cytotoxicity was tested against cancer and normal cell lines. CA inhibition, cell cycle effects, apoptosis induction, and protein expression changes were evaluated. Molecular docking and ADMET predictions assessed binding and drug-like properties.

**Results and Discussion:** **Pyr** showed selective cytotoxicity toward cancer cells and potent CA IX inhibition. It induced G0/G1 arrest, apoptosis, and modulated p53, Bax, and Bcl-2 levels. Docking confirmed strong CA IX binding, and ADMET analysis indicated good oral bioavailability. These results support **Pyr** as a promising anticancer candidate.

## 1 Introduction

Cancer remains one of the most challenging health conditions worldwide, characterized by uncontrolled cell growth, invasion into surrounding tissues, and potential metastasis to distant organs (Wikipedia, 2024). It affects millions of lives annually, highlighting the urgent need for effective therapeutic strategies (WHO, 2024). Advances in cancer biology have paved the way for developing anticancer agents targeting various cellular and molecular mechanisms involved in tumor initiation, progression, and metastasis (Doostmohammadi et al., 2024). Current treatments, including chemotherapy, targeted therapy, and immunotherapy, often aim to disrupt specific pathways critical to cancer survival and growth (Yang et al., 2024; P. Inc, 2024; Tilsed et al., 2022). Despite significant progress, the emergence of drug resistance and adverse side effects highlight the need for novel therapeutic targets and agents with improved efficacy and safety profiles (Targeted Cancer Therapy, 2024; Tian et al., 2024).

Among the emerging molecular targets in cancer therapy, carbonic anhydrases (CAs) have gained attention due to their critical role in maintaining the pH balance within tumor cells and their microenvironment (Pastorekova and Gillies, 2019). Carbonic anhydrases are zinc metalloenzymes with 15 known isoforms in humans, of which CA IX and CA XII are overexpressed in hypoxic tumor cells (Baranauskienė et al., 2019). These isoforms play a pivotal role in regulating intracellular and extracellular pH by catalyzing the reversible hydration of carbon dioxide to bicarbonate and protons, enabling cancer cells to thrive in acidic and hypoxic conditions (Sedlakova et al., 2014). By supporting an acidic extracellular environment, CA IX and CA XII facilitate tumor progression, invasion, and immune evasion. Consequently, carbonic anhydrase inhibitors (CAIs) have emerged as promising anticancer agents, selectively targeting tumor-associated isoforms to disrupt these critical processes (Merkx et al., 2021). Recent studies have demonstrated the potential of CAI to inhibit tumor growth, enhance the effectiveness of conventional chemotherapeutics, and overcome resistance mechanisms (Kalinin et al., 2021).

SLC-0111, a ureido-substituted benzenesulfonamide, is a potent inhibitor of CA IX, disrupting pH regulation in tumor cells and impairing their survival and invasiveness (MedChemExpress, 2024). Preclinical studies have demonstrated that SLC-0111 enhances the efficacy of chemotherapeutic agents such as cisplatin in head and neck squamous carcinoma models, leading to reduced tumor growth and invasion (Sarnella et al., 2022). Additionally, SLC-0111 has progressed to Phase I clinical trials, where it was evaluated for safety and tolerability in patients with advanced solid tumors (McDonald et al., 2020).

**FIGURE 1:**
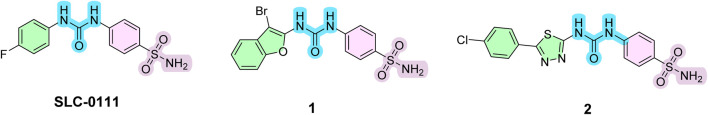
Examples of important inhibitors of tumor-associated carbonic anhydrase isoforms.

Building upon the success of SLC-0111, researchers have developed various analogs to enhance selectivity and potency against tumor-associated carbonic anhydrase (CA) isoforms (Angeli et al., 2023). For instance, benzofuran-based derivatives have been synthesized, exhibiting selective inhibition of CA IX and CA XII (Figure 1) and demonstrating promising anticancer activity, with compound **1** displaying significant inhibitory activity against CA IX with a Ki value of 7.1 nM (Shaldam et al., 2021). Similarly, thiazole and thiadiazole-based analogs have shown substantial inhibitory activity against tumor-associated CA isoforms with thiadiazole analog 2 exhibited remarkable Ki values of 7.9 and 9.9 nM for CA IX and CA XII inhibition, respectively (Abo-Ashour et al., 2019). These derivatives have effectively suppressed tumor cell proliferation and migration, underscoring their promise as therapeutic agents in cancer treatment by targeting carbonic anhydrases.

In parallel with the development of small molecule inhibitors targeting CA IX, immunotherapeutic strategies have also emerged as promising approaches for the treatment of CA IX-expressing tumors. Notably, bi-specific adapter molecules capable of engaging universal chimeric antigen receptor T (CAR-T) cells have been designed to facilitate the selective targeting of CA IX-positive tumor cells (Low et al., 2024). These adapters act as molecular bridges that redirect CAR-T cells to tumor sites while providing an additional layer of control, potentially reducing off-tumor toxicity and enhancing safety profiles. By enabling the specific recognition and elimination of hypoxic, CA IX-expressing tumor cells, these bi-specific adapters offer a novel immunotherapeutic avenue that could complement the effects of CA IX inhibitors and broaden the therapeutic landscape for solid tumors characterized by hypoxia and acidosis.

**FIGURE 2:**
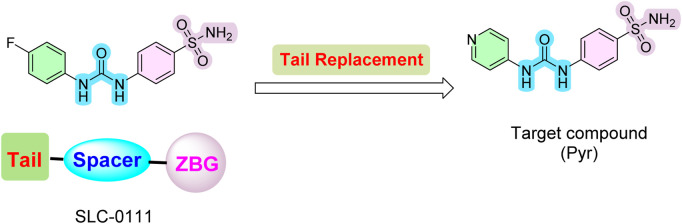
Design of novel SLC-0111 analog (**Pyr**).

Inspired by these findings on SLC-0111 and its related analogs, we employed a tail approach to design and synthesize a new analog of SLC-0111, incorporating a 4-pyridyl moiety linked with sulfanilamide as the zinc-binding group. This analog was evaluated for its anticancer properties and inhibitory activity against four CA isoforms: CA I, II, IX, and XII. The design retained the zinc-binding sulfonamide motif and urea linker from SLC-0111 while introducing a 4-pyridyl moiety as a tail to enhance interactions with the hydrophobic region of the CA active site (Figure 2).

## 2 Results and discussion

### 2.1 Chemistry

**SCHEME 1:**
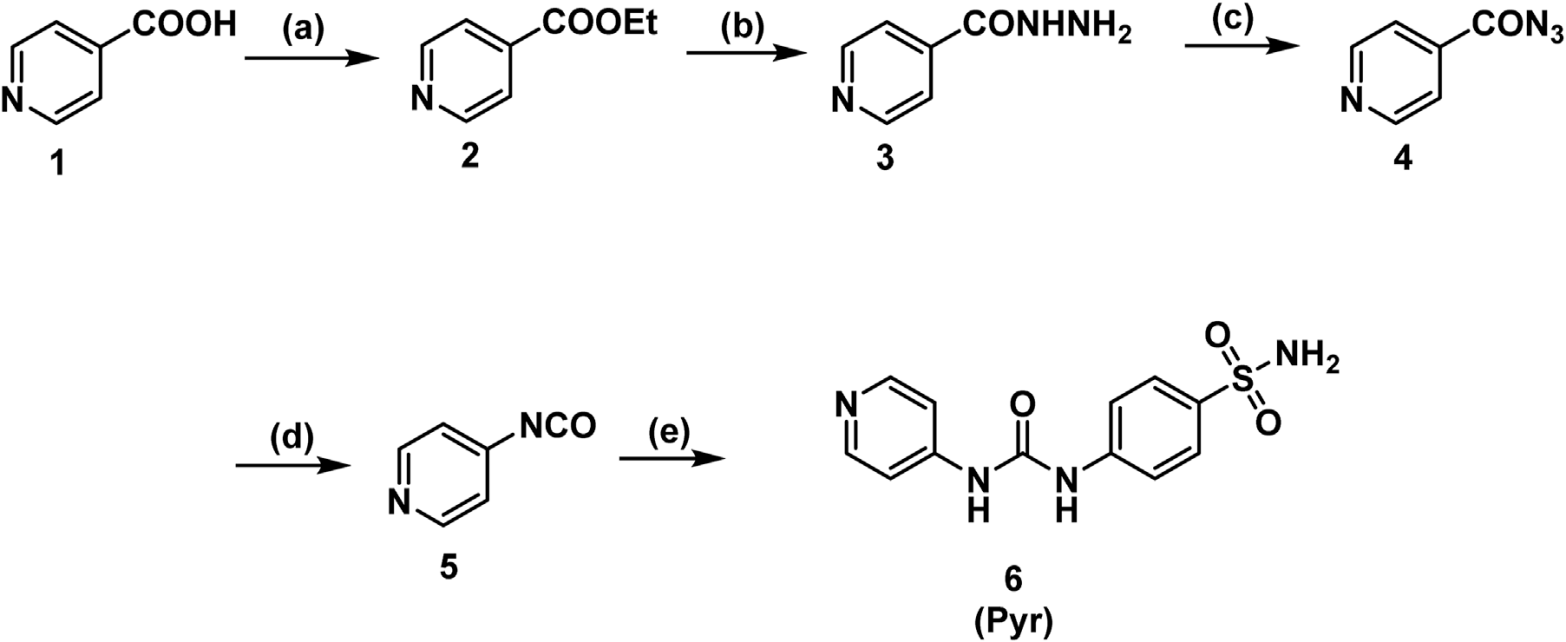
Synthesis of the target compound (**Pyr**). Reagents and Conditions: (a) EtOH, H_2_SO_4_, reflux, 8 h; (b) hydrazine hydrate, EtOH, reflux, 18 h; (c) NaNO_2_, HCl, 0°C, 1 h; (d) toluene, reflux, 1 h; (e) sulfanilamide, toluene, reflux, 2h.

Scheme 1 illustrates the stepwise synthesis of a sulfonamide-based urea derivative starting from pyridine-4-carboxylic acid (isonicotinic acid). The synthesis begins with the Fischer esterification of isonicotinic acid, converting its carboxylic acid group into an ethyl ester using absolute ethanol in the presence of concentrated sulfuric acid as a catalyst. In the second step, the ethyl ester reacts with hydrazine hydrate, replacing the ethoxy group with a hydrazino group to form the corresponding hydrazide (Isoniazid). The hydrazide is then subjected to diazotization using sodium nitrite in the presence of hydrochloric acid, leading to the formation of the acyl azide intermediate. This reactive intermediate is key to the next transformation, the Curtius rearrangement, which is induced by heating the acyl azide in toluene. During this rearrangement, the azide undergoes nitrogen gas evolution (N_2_) and acyl migration, forming an isocyanate intermediate. The highly reactive isocyanate is then reacted with sulfanilamide under heating in toluene to yield the final sulfonamide-based urea derivative **Pyr**. This step involves a nucleophilic attack by the amine group of sulfanilamide on the electrophilic carbon of the isocyanate group, forming the desired product.

The final compound, **Pyr**, was characterized using ^1^H-NMR, ^13^C-NMR, and elemental analysis. The ^1^H-NMR spectrum confirms the structure of the compound with distinct peaks corresponding to its key protons. The urea moiety is represented by two singlets at 8.97 ppm and 9.10 ppm, confirming the presence of the urea hydrogens. The protons of the pyridine ring appear as two doublets at 8.34 ppm and 7.44 ppm, consistent with the expected splitting for this system. Similarly, the protons of the para-substituted benzene ring are represented by two doublets at 7.62 ppm and 7.73 ppm. The NH_2_ group of the sulfonamide appears as a singlet at 7.24 ppm. In the ^13^C-NMR spectrum, eight peaks are observed, consistent with the distinct carbon atoms in the molecule. Four peaks are assigned to the benzene ring carbons, while three peaks correspond to the pyridine ring carbons. The carbonyl carbon of the urea group is confirmed by a characteristic peak at 150.40 ppm, supporting the successful formation of the urea moiety. These findings from the NMR data align well with the proposed structure of the target compound.

### 2.2 Biological evaluation

#### 2.2.1 Cell viability assay

**FIGURE 3:**
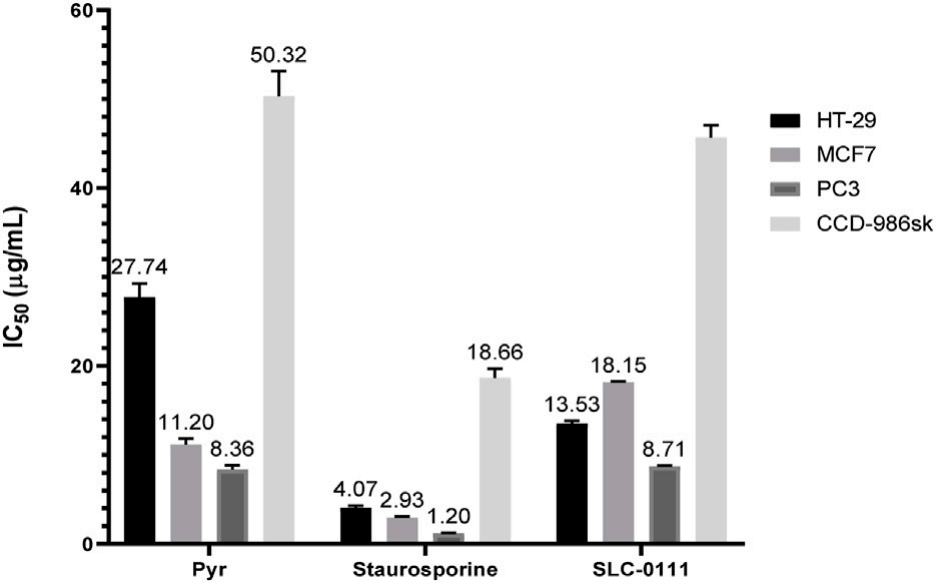
IC_50_ values (µg/mL) for **Pyr** and Staurosporine against HT-29, MCF7, PC3 cancer cells, and CCD-986sk normal cells. **Pyr** exhibited moderate cytotoxicity with better selectivity for cancer cells, while Staurosporine showed higher potency but less selectivity. Data are mean ± SEM.

Many sulfonamide-based compounds have shown promising cytotoxic activities (Güleç et al., 2024a; Güleç et al., 2024b) against various cancer cell lines. Accordingly, the cytotoxic activity of **Pyr** was evaluated against HT-29, MCF7, and PC3 cancer cell lines, as well as the normal CCD-986sk cell line. Based on the cell viability assay results shown in Figure 3, **Pyr** exhibited moderate cytotoxicity against the tested cancer cell lines (HT-29, MCF7, and PC3) with IC_50_ values of 27.74, 11.20, and 8.36 µg/mL, respectively, while showing significantly lower toxicity toward the normal cell line CCD-986sk (IC_50_ = 50.32 µg/mL), indicating good selectivity. Compared to the reference SLC-0111, Pyr demonstrated superior cytotoxicity against the MCF7 cell line (IC_50_ = 11.20 µg/mL vs. 18.15 µg/mL for SLC-0111) and comparable activity against the PC3 cell line (IC_50_ = 8.36 µg/mL vs. 8.71 µg/mL), whereas SLC-0111 exhibited better potency against the HT-29 cell line (IC_50_ = 13.53 µg/mL vs. 27.74 µg/mL for Pyr). Regarding safety, Pyr also showed slightly lower toxicity toward normal CCD-986sk cells (IC_50_ = 50.32 µg/mL) compared to SLC-0111 (IC_50_ = 45.70 µg/mL), highlighting its favorable selectivity profile. In contrast, the reference drug Staurosporine is significantly more potent across all cell lines, with much lower IC_50_ values for HT-29 (4.07 µg/mL), MCF7 (2.93 µg/mL), and PC3 (1.20 µg/mL). Still, it also exhibits considerable toxicity to normal cells (IC_50_ = 18.66 µg/mL), reflecting poor selectivity. These findings suggest that while Staurosporine is highly effective, its lack of selectivity limits its therapeutic potential. By contrast, with its selective cytotoxicity, superior or comparable efficacy to SLC-0111 in certain cancer cell lines, and reduced toxicity to normal cells, Pyr presents a more favorable profile for further development as an anti-cancer agent.

#### 2.2.2 Evaluation of carbonic anhydrase I, II, IX, and XII inhibition

Sulfonamides are well-known inhibitors of carbonic anhydrase (CA) enzymes (Buza et al., 2024; Buza et al., 2023; Kakakhan et al., 2023) and have been widely studied for their potential therapeutic applications, particularly in cancer and other diseases. Given their significance as CA inhibitors, we evaluated **Pyr**’s inhibitory activity against various human carbonic anhydrase isoforms, specifically CA I, CA II, CA IX, and CA XII. The results in Table 1 present the IC_50_ values (µg/mL) of **Pyr** and the reference drugs acetazolamide (AAZ) and SLC-0111 against carbonic anhydrase (CA) isoforms I, II, IX, and XII. **Pyr** showed moderate inhibition of CA I (20.29 ± 0.92 µg/mL), CA II (0.569 ± 0.03 µg/mL) and CA XII (2.97 ± 0.17 µg/mL) with higher potency against the cancer-related isoform CA IX (0.399 ± 0.02 µg/mL). In comparison, AAZ, a standard carbonic anhydrase inhibitor, exhibited significantly lower IC_50_ values across all isoforms, with exceptional potency against CA II (0.153 ± 0.01 µg/mL), CA IX (0.105 ± 0.01 µg/mL), and CA XII (0.029 ± 0.001 µg/mL). Similarly, SLC-0111, a selective inhibitor of tumor-associated carbonic anhydrases, showed strong inhibition of CA IX (0.048 ± 0.006 µg/mL) and CA XII (0.096 ± 0.008 µg/mL). These results suggest that while **Pyr** is less potent than AAZ and SLC-0111 overall, it demonstrated selective inhibition of CA IX, a tumor-associated isoform, indicating its potential as a targeted inhibitor for cancer-related applications.

**TABLE 1:** IC_50_ values (µg/mL) of **Pyr** and acetazolamide (AAZ) against CA isoforms I, II, IX, and XII. Data are expressed as mean ± SEM.

Compound	CA inhibition IC_50_ (μg/mL) ±SEM							
	CA I	R^2^	CA II	R^2^	CA IX	R^2^	CA XII	R^2^
Pyr	20.29 ± 0.92	0.9406	0.569 ± 0.03	0.9697	0.399 ± 0.02	0.9816	2.97 ± 0.17	0.9645
AAZ	0.367 ± 0.02	0.9782	0.153 ± 0.01	0.9615	0.105 ± 0.01	0.972	0.029 ± 0.001	0.9315
SLC-0111	1.36 ± 0.07	0.9935	0.498 ± 0.04	0.9767	0.048 ± 0.006	0.9642	0.096 ± 0.008	0.9873

#### 2.2.3 Cell cycle analysis

**FIGURE 4:**
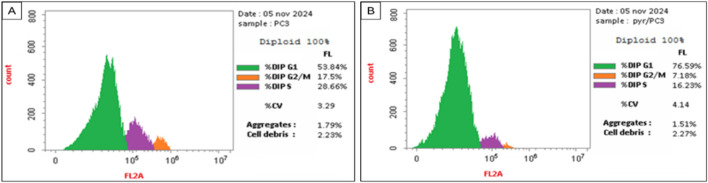
Effect of DMSO (Control, **(A)**) and **Pyr** (IC_50_, 8.63 μg/mL, **(B)**) on cell accumulation across various phases of the cell cycle of prostate PC3 cancer cells.

The data in Table 2 and Figure 4 illustrate the impact of **Pyr** on the cell cycle distribution of PC3 cells compared to untreated cells (treated with DMSO as a control). Treatment of PC3 cells with **Pyr** significantly increased the percentage of cells in the G0-G1 phase (76.59%) compared to the control (53.84%), indicating a G0-G1 phase arrest. Correspondingly, the percentage of cells in the S phase decreased notably in **Pyr**-treated cells (16.23%) compared to the control (28.66%), suggesting inhibition of DNA synthesis and progression to the S phase. Furthermore, the percentage of cells in the G2/M phase was reduced in **Pyr**-treated cells (7.18%) compared to the control (17.50%), indicating impaired transition to mitosis. These results suggest that **Pyr** effectively induces cell cycle arrest at the G0-G1 phase in PC3 cells, thereby inhibiting cell proliferation.

**TABLE 2:** Effect of **Pyr** derivative on PC3 cell cycle distribution compared to DMSO-treated cells.

Compound	DNA content		
	% G0-G1	% S	% G2/M
Pyr/PC3	76.59	16.23	7.18
DMSO/PC3	53.84	28.66	17.50

#### 2.2.4 Apoptosis assay

**FIGURE 5:**
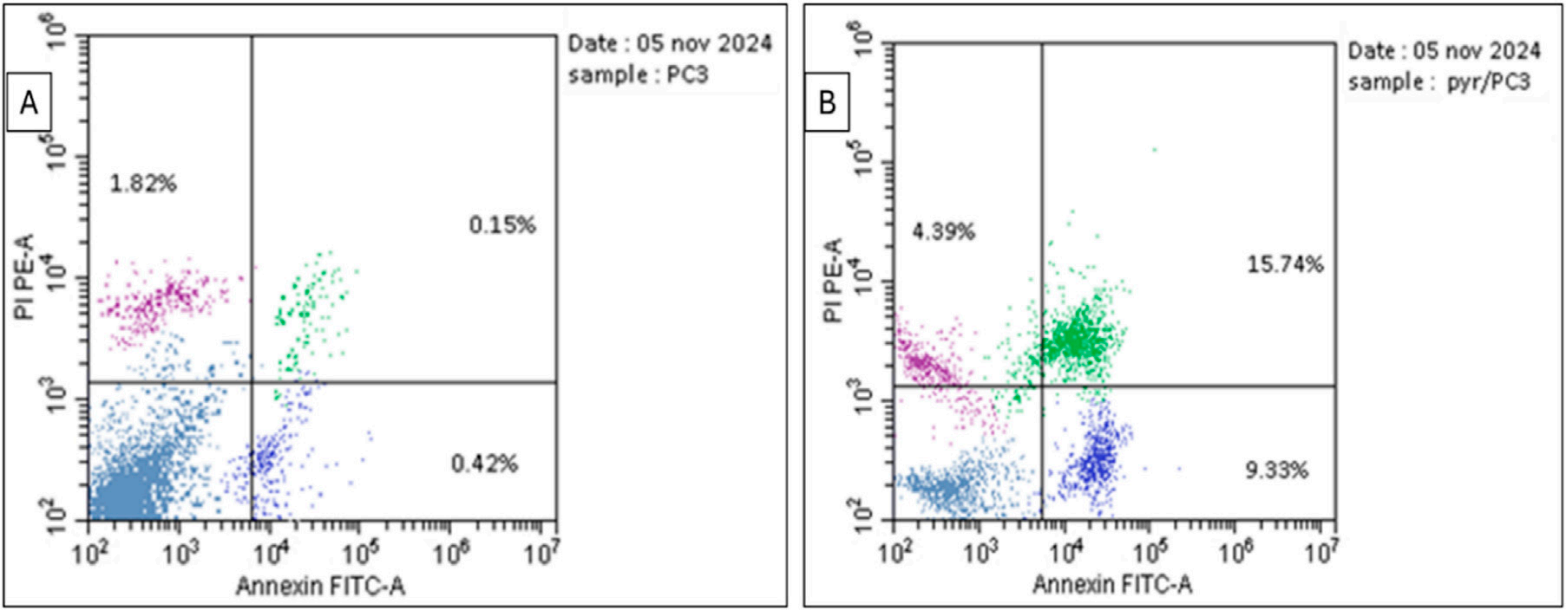
Flow cytometric dot plot for prostate PC3 cancer cells treated with **Pyr** and untreated cells following Annexin V-FITC/PI staining. **(A)** shows untreated cells, while **(B)** shows PC3 cells treated with **Pyr** at IC_50_ (8.63 μg/mL). The four quadrants are labeled as follows: Lower Left (LL) for viable cells, Lower Right (LR) for early apoptotic cells, Upper Left (UL) for necrotic cells, and Upper Right (UR) for late apoptotic cells.

The data in Table 3 and Figure 5 highlight the effect of **Pyr** on apoptosis and necrosis in PC3 cells compared to untreated cells (DMSO-treated cells). Treatment of PC3 cells significantly increased total apoptosis to 29.46% compared to 2.39% in the untreated cells. This increase is driven by both early apoptosis (9.33%) and late apoptosis (15.74%), indicating that **Pyr** effectively triggers the apoptotic pathway in PC3 cells. Necrosis levels were also slightly elevated in **Pyr**-treated cells (4.39%) compared to the control (1.82%), though apoptosis remains the predominant mode of cell death. These findings suggest that **Pyr** induces substantial apoptotic cell death in PC3 cells, with minimal necrotic activity, highlighting its potential as an anti-cancer agent targeting apoptosis.

**TABLE 3:** Effect of **Pyr** derivative on apoptosis and necrosis in PC3 cells compared to DMSO-treated cells.

Compound	Apoptosis			Necrosis
	Total	Early	Late	
Pyr/PC3	29.46	9.33	15.74	4.39
DMSO/PC3	2.39	0.42	0.15	1.82

#### 2.2.5 Effects of Pyr on p53, Bax and Bcl-2 protein expression levels

The effect of **Pyr** on the expression of pro-apoptotic (p53, Bax) and anti-apoptotic (Bcl-2) proteins in PC3 cells compared to untreated cells was studied. As shown in Table 4, **Pyr** treatment markedly increased the expression levels of p53 and Bax, while simultaneously reducing Bcl-2 levels when compared to the DMSO-treated control group. Specifically, **Pyr** treatment elevated p53 protein concentration to 1461.33 ± 10.00 pg/mL, corresponding to a 9.66-fold increase relative to control cells (151.33 ± 3.33 pg/mL). This substantial upregulation of p53 suggests the activation of a p53-dependent apoptotic pathway, which is known to play a critical role in tumor suppression through cell cycle arrest and apoptosis induction. Concurrently, Bax, a key pro-apoptotic member of the Bcl-2 family, was significantly increased to 477.27 ± 18.54 pg/mL (5.85-fold), supporting the pro-apoptotic effect of **Pyr** by promoting mitochondrial outer membrane permeabilization and the subsequent release of apoptogenic factors. In contrast, Bcl-2, an anti-apoptotic protein that antagonizes Bax and preserves mitochondrial integrity, was notably decreased following **Pyr** treatment, reaching 8.88 ± 0.30 pg/mL, which represents a 0.409-fold reduction compared to control (21.68 ± 0.70 pg/mL). This decline in Bcl-2 expression further emphasizes the potential of **Pyr** to disrupt cellular survival pathways, favoring apoptosis over proliferation in PC3 cells.

**TABLE 4:** Effects of **Pyr** on p53, Bax, and Bcl-2 protein expression levels in PC3 cells.

Compound	P53		Bax		Bcl-2	
	Conc (pg/mL)	Fold change	Conc (pg/mL)	Fold change	Conc (pg/mL)	Fold change
Pyr/PC3	1461.33 ± 10.00	9.66	477.27 ± 18.54	5.85	8.88 ± 0.3	0.409
DMSO/PC3	151.33 ± 3.33	1	81.49 ± 3.16	1	21.68 ± 0.7	1

#### 2.2.6 Effects of Pyr on caspase-3 and caspase-9 activities

To further explore the apoptotic mechanism induced by the synthesized compound **Pyr**, the activities of caspase-3 and caspase-9—two critical executioners of the intrinsic apoptosis pathway—were assessed in PC3 cells. As presented in Table 5, **Pyr** treatment led to a significant increase in the activities of both caspase-3 and caspase-9 when compared to the DMSO-treated control. The concentration of caspase-3 in Pyr-treated cells reached 573.19 ± 22.27 pg/mL, reflecting an 8.55-fold elevation relative to the control (67.02 ± 2.60 pg/mL). This notable increase underscores the activation of downstream apoptotic processes, as caspase-3 is a key effector protease responsible for the cleavage of vital cellular substrates during apoptosis. Similarly, the concentration of caspase-9, an essential initiator of the mitochondrial apoptosis pathway, was significantly enhanced following **Pyr** treatment, rising to 41.82 ± 0.95 ng/mL, which corresponds to an 8.97-fold increase compared to control cells (4.66 ± 0.41 ng/mL). The activation of caspase-9 indicates the involvement of the intrinsic (mitochondrial) pathway, consistent with the observed modulation of upstream regulators, including p53, Bax, and Bcl-2.

**TABLE 5:** Effects of **Pyr** on caspase-3 and caspase-9 activities in PC3 cells.

Compound	Caspase 3		Caspase-9	
	Conc (pg/mL)	Fold change	Conc (ng/mL)	Fold change
Pyr/PC3	573.19 ± 22.27	8.55	41.82 ± 0.95	8.97
DMSO/PC3	67.02 ± 2.6	1	4.66 ± 0.41	1

### 2.3 Molecular modeling studies

#### 2.3.1 DFT calculations

##### 2.3.1.1 Geometrical structure and frontier molecular orbitals (FMOs)

**FIGURE 6:**
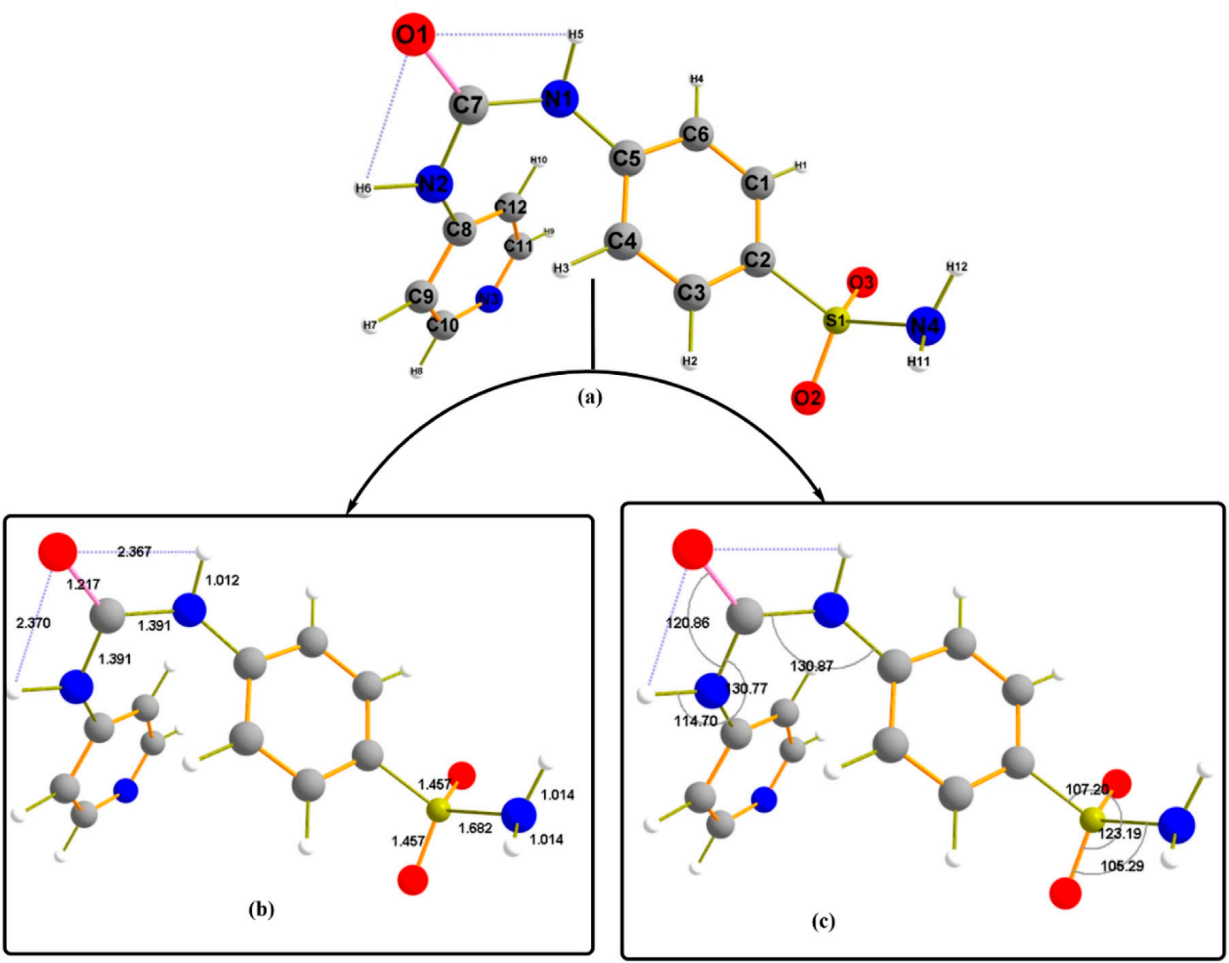
The geometrical structure of the designed compound **Pyr (a)** labeled with **(b)** bond lengths, **(c)** bond angles.

Figure 6 illustrates the studied geometrical structural convergence of **Pyr** in the ground state using the same theoretical level. To better understand the conformational behavior of the structure under consideration, it is feasible to talk about two essential structural properties: bond lengths and bond angles. The compound’s difference in planarity is explained by the face direction of the two aromatic rings, directing the sulphonyl amino group (SO_2_NH_2_) away from the plane of the molecule. Furthermore, an important hint to the bent structure found during optimization is provided by the CO group that considered the center of molecule bending and forms two H-bonds with the N1H5 and N2H6 with estimated bond distances of 2.367 Å and 2.370 Å, respectively. This feature may contribute to the structural stability of **Pyr**. Given that the covalent link between N and H is 1.012 Å longer than the usual bond length (Sayed and Abdelrehim, 2022), the observed N5-H and N6-H bond length validates the strength of the H-bond formation. The optimized structure’s bond angle values allow for elaborating the atoms’ planarity behavior. Bond angle values evaluated the similarity in the environment surrounding the molecule, as in the case of C5-N1-C7 and C7-N2-C8 with 130.77^°^.

**FIGURE 7:**
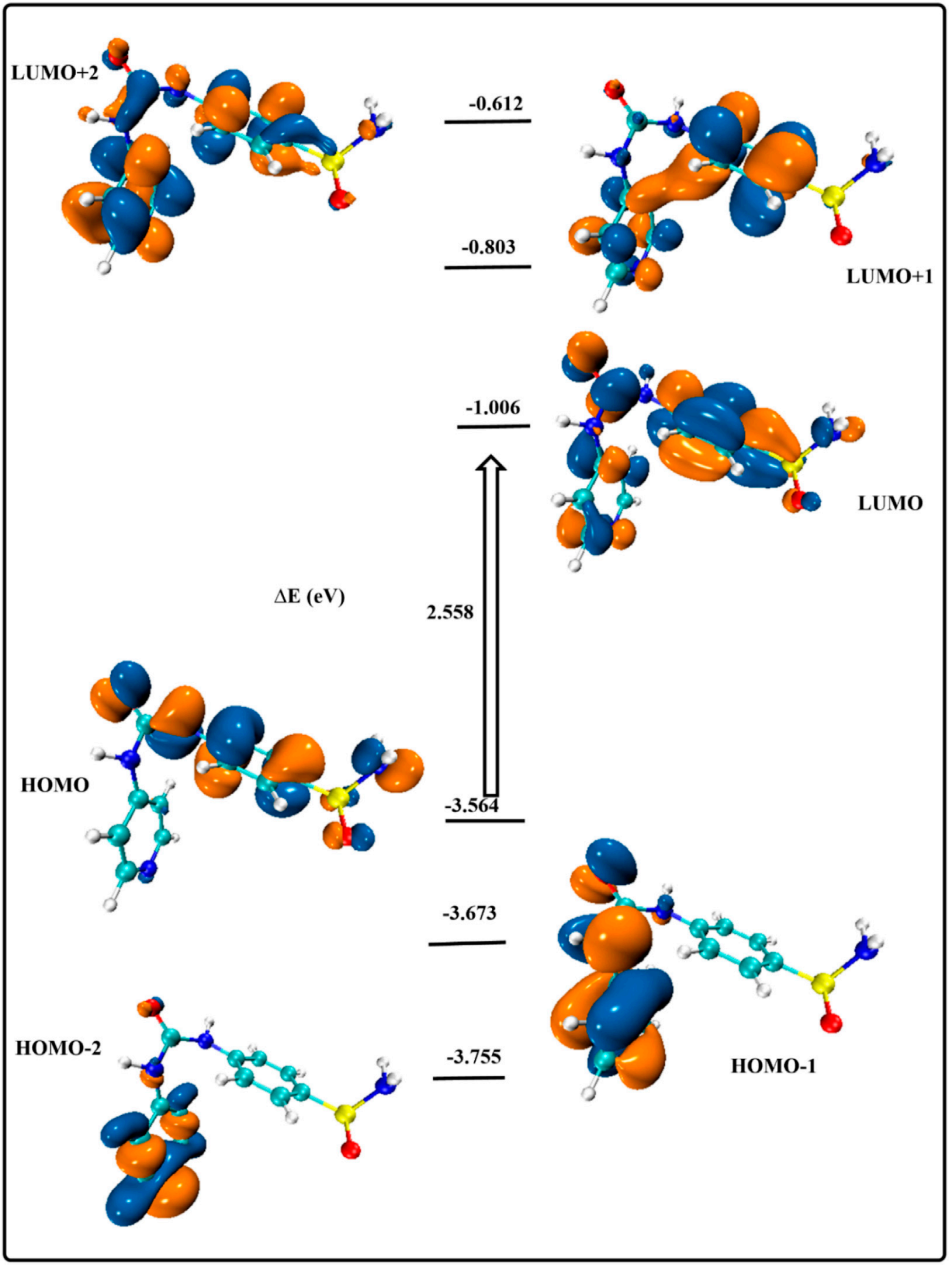
Energy excitation levels with energy values (eV).

To predict the stability and reactivity of different molecules, it is essential to study the features of compounds’ frontier molecular orbitals (FMOs) (Abdelrehim and El-Sayed, 2020). Figure 7 displays the energy distribution of the most significant molecular orbitals for the gaseous optimized structures (HOMO-2, HOMO-1, HOMO, LUMO, LUMO+1, and LUMO+2). The stability of the current heterocyclic complex can be predicted using the amplitude of the energy gap (∆E = 2,558 eV) between HOMO and LUMO levels, a crucial parameter in the study of stability and reactivity (Chen et al., 2021). The orbital contribution at all examined levels was mostly observed throughout the proposed molecule, providing additional evidence for the stability of both ground and excited states. The orbital contribution in the FMOs mostly occurs around SO_2_ and CONH groups, leading to the concept of successive donor-acceptor interactions in the excited states.

##### 2.3.1.2 UV–Vis electronic spectra by TD-DFT method

**FIGURE 8:**
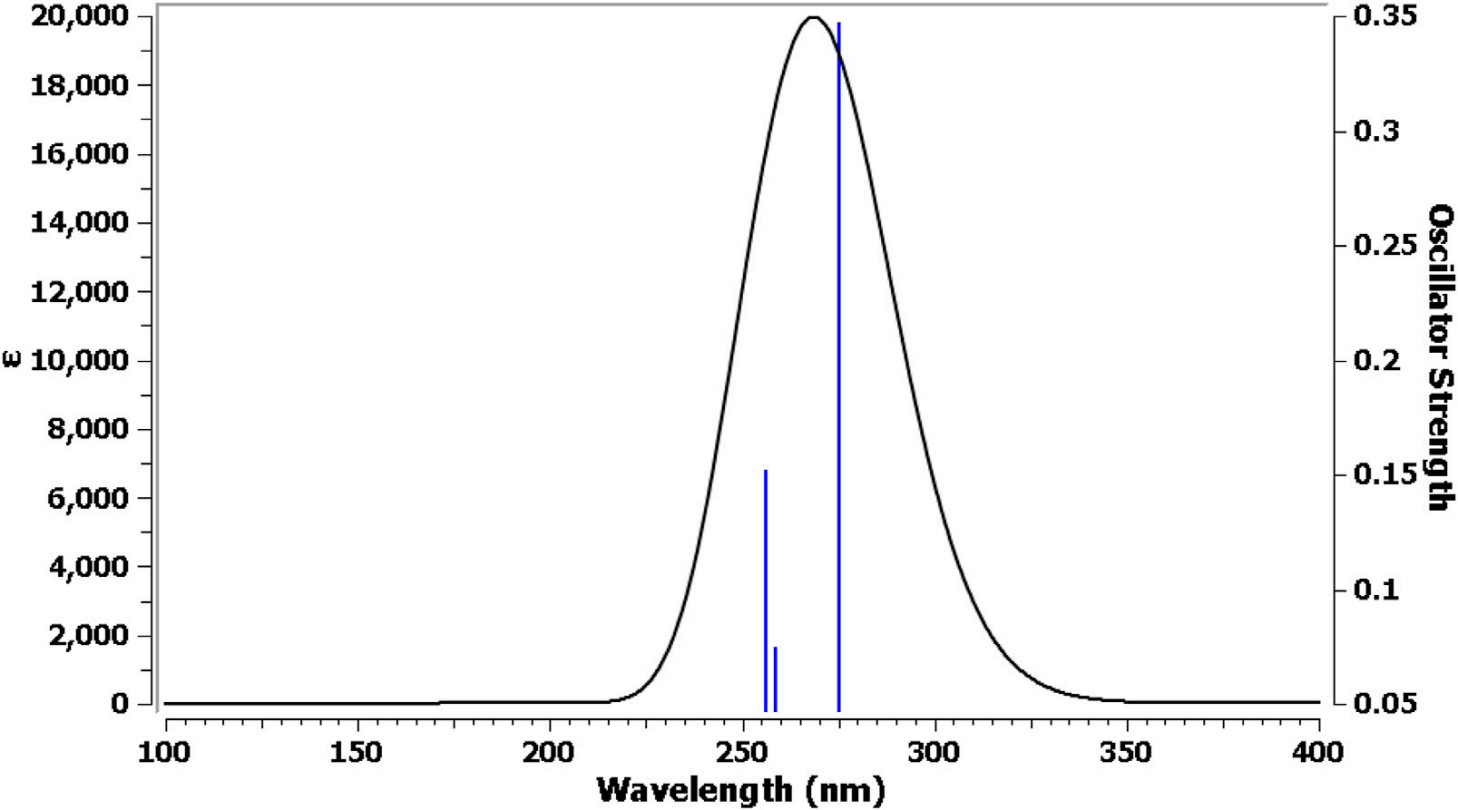
UV–Vis electronic absorption spectra for **Pyr**.

The TDDFT and CPCM solvation model was used to characterize the electrical behavior of **Pyr**. The default Gaussian 09 parameters were used for the TD-DFT calculations. The software settings were adjusted for Nstate = 6 to study six states. In Figure 8, three transition bands are shown as lines. The LOG file of the Gaussian calculation shows a singlet strong absorption band for the first transition, which is the n-π* transition. With a 64.3% transition contribution, an excitation energy of 4.505 eV, and a maximum wavelength (λ_max_) of 275 nm, the orbital contribution comprises HOMO→LUMO excitation with n-π type of electronic transition. The oscillator strength (F) revealed the strong ability of electronic achievement to LUMO excited state, where its value is higher in the first transition (0.347). Table 6 shows that while the other transitions were anticipated from HOMO→LUMO+1and from HOMO-1→LUMO with comparable contribution %, their F values differ based on the ability of electron transition from one level to another.

**TABLE 6:** Excitation energies, maximum wavelengths, oscillator strengths, and % orbital contribution for **Pyr**.

Spectral line number	Excitation energy (eV)	λ_max_ (nm)	F	Type of transition	% orbital contribution
1	4.505	275	0.347	HOMO→LUMO	64.3
2	4.793	259	0.074	HOMO→LUMO+1	54
3	4.839	256	0.151	HOMO-1→LUMO	64.8

##### 2.3.1.3 Electron localization function (ELF)

**FIGURE 9:**
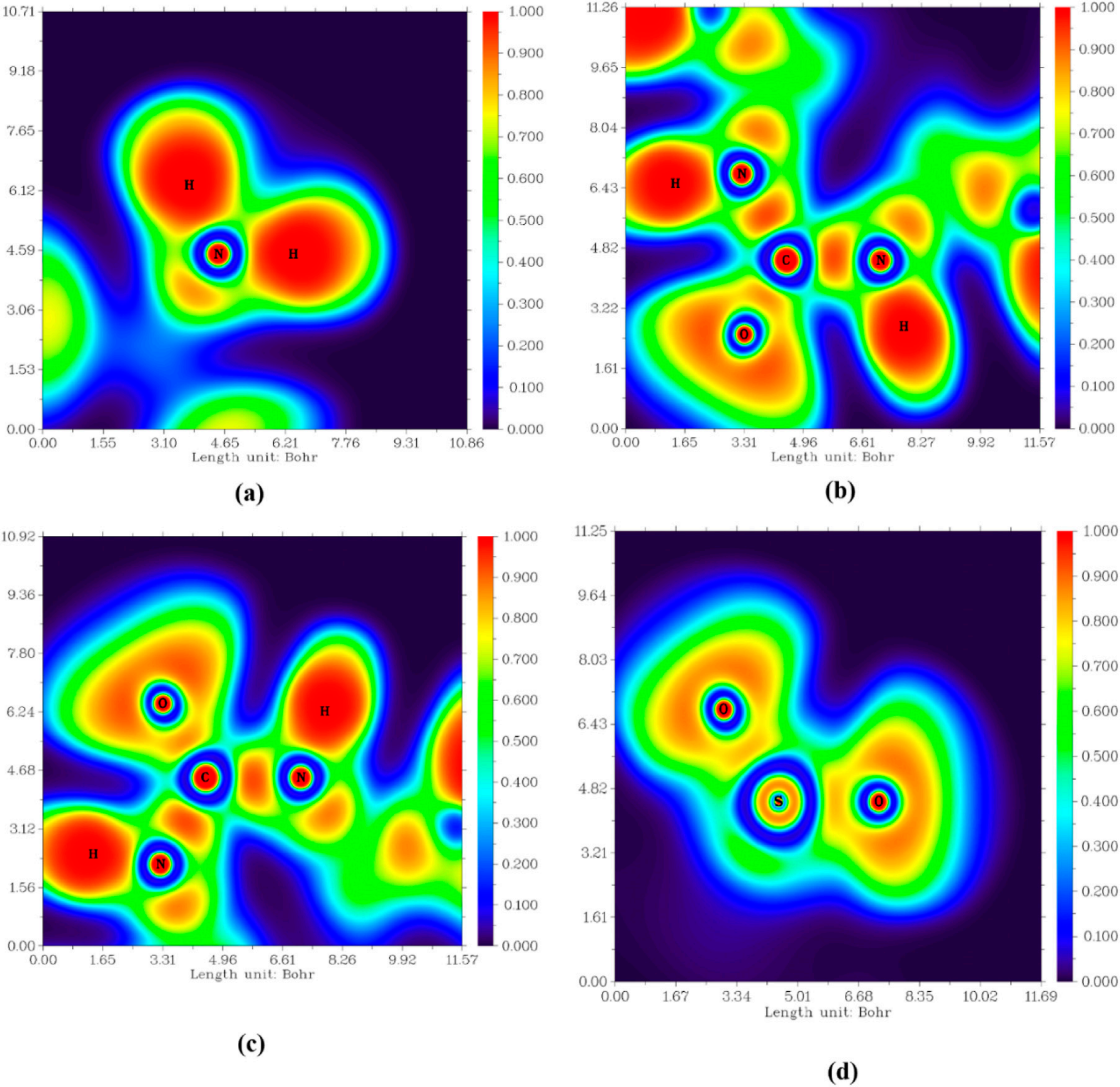
Electron localization function (ELF) colored map of **Pyr (a)** H11-N4-H12, **(b)** N1-C7-N2, **(c)** N1-C7-O1, and **(d)** O2-S1-O3.

The electron localization function (ELF) explores the empirical concepts of electron localization, especially the localization of electron pairs, in the spirit of Lewis structures. An Electron Localization Function (ELF) in atomic space designates a point where electron confinement and bond type are known (Emara et al., 2023). One of the most important two-dimensional planes offers details on the type of bond in three distinct planes: H11-N4-H12, N1-C7-N2, N1-C7-O1, and O2-S1-O3. All the atoms of interest are exhibited at the same plane, as shown in Figure 9. The selected planes estimate a localized electronic area (red color scale) between C7 and N atoms (both N1 and N2) and the same environment (appear in the same plane). At the same time, the plane of the amino group exhibits unusual behavior as no additional atoms are observed in this plane with slightly localized electronic density in the bond of interaction between N4 and S1 (not of the same plane). So, the electron localization between N and the second coordinated atom located in the other plane is predicted by this finding, completing the geometrical structure with the optimum conditions. The map of electronic deformation around O1 decreases the electronic localization between it and C7, as displayed in Figure 9c. Another reason for this deformation is a prediction of H-bond formation between O and the other surrounded H atoms. The bond nature between S and O atoms in the O2-S1-O3 is exhibited to be less strong due to the delocalization of electrons around the two O atoms.

##### 2.3.1.4 Molecular electrostatic potential (MEP)

**FIGURE 10:**
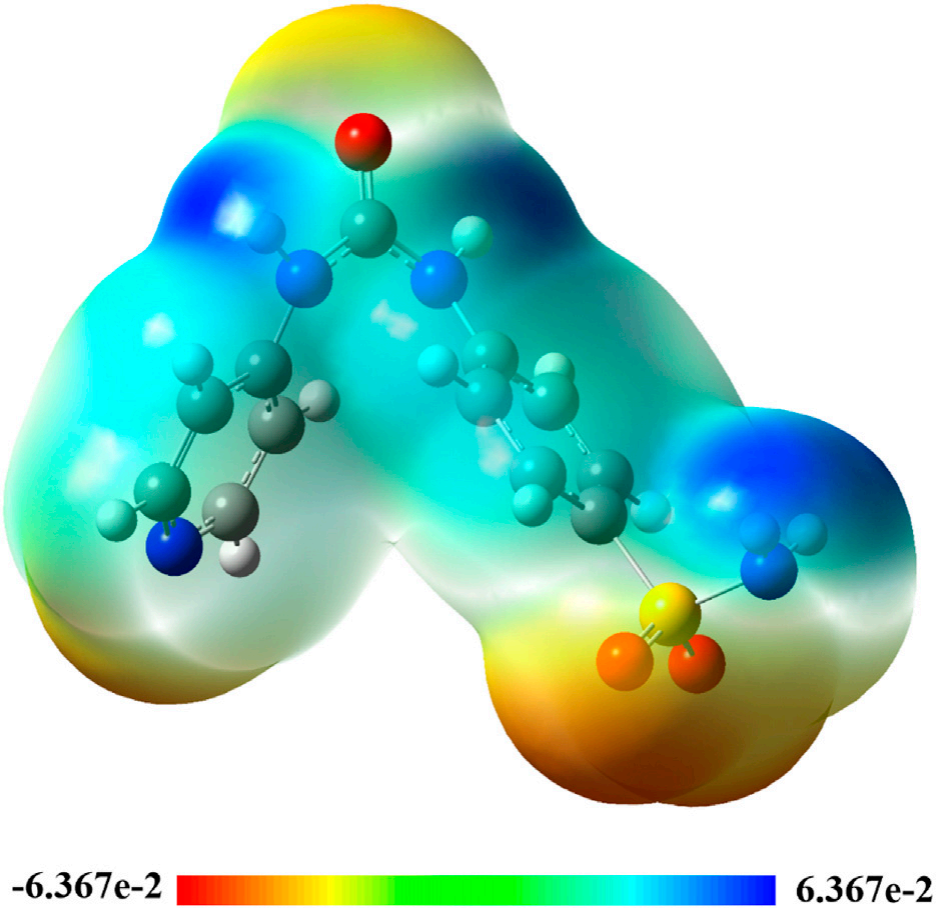
3D-colored map of the molecular electrostatic potential of **Pyr**.

The molecular electrostatic potential (MEP) 3D map is used to analyze the electronegativities of atomic locations on molecules. This topological index helps explain molecular contacts and recognition processes since long-range interactions are mostly caused by electrostatic forces (Issa et al., 2024). The structures of the studied compounds were visually evaluated using colors such as red, orange, yellow, green, and blue to identify potential sites of electrophilic or nucleophilic assaults (Figure 10). The following order of colors represents the decreasing potential for each atomic site: orange, red, green, yellow, and blue. As a result, a red zone indicates an electron-rich site and a blue zone indicates an electron-deficient site. More precisely, it was predicted that all oxygen atom sites in **Pyr** would have the maximum electronic richness, with the remaining portion of the molecule striving to reach a neutral state and only having a small density of π-electrons of the phenyl ring (yellow color scale). This discrepancy is believed to direct a significant electron donation from the nucleophilic O site to the electron acceptor of electrophilic sites, mainly including amino group hydrogen atoms (primary and secondary groups).

##### 2.3.1.5 Reduced density gradient/non-covalent-interactions (RDG/NCI)

**FIGURE 11:**
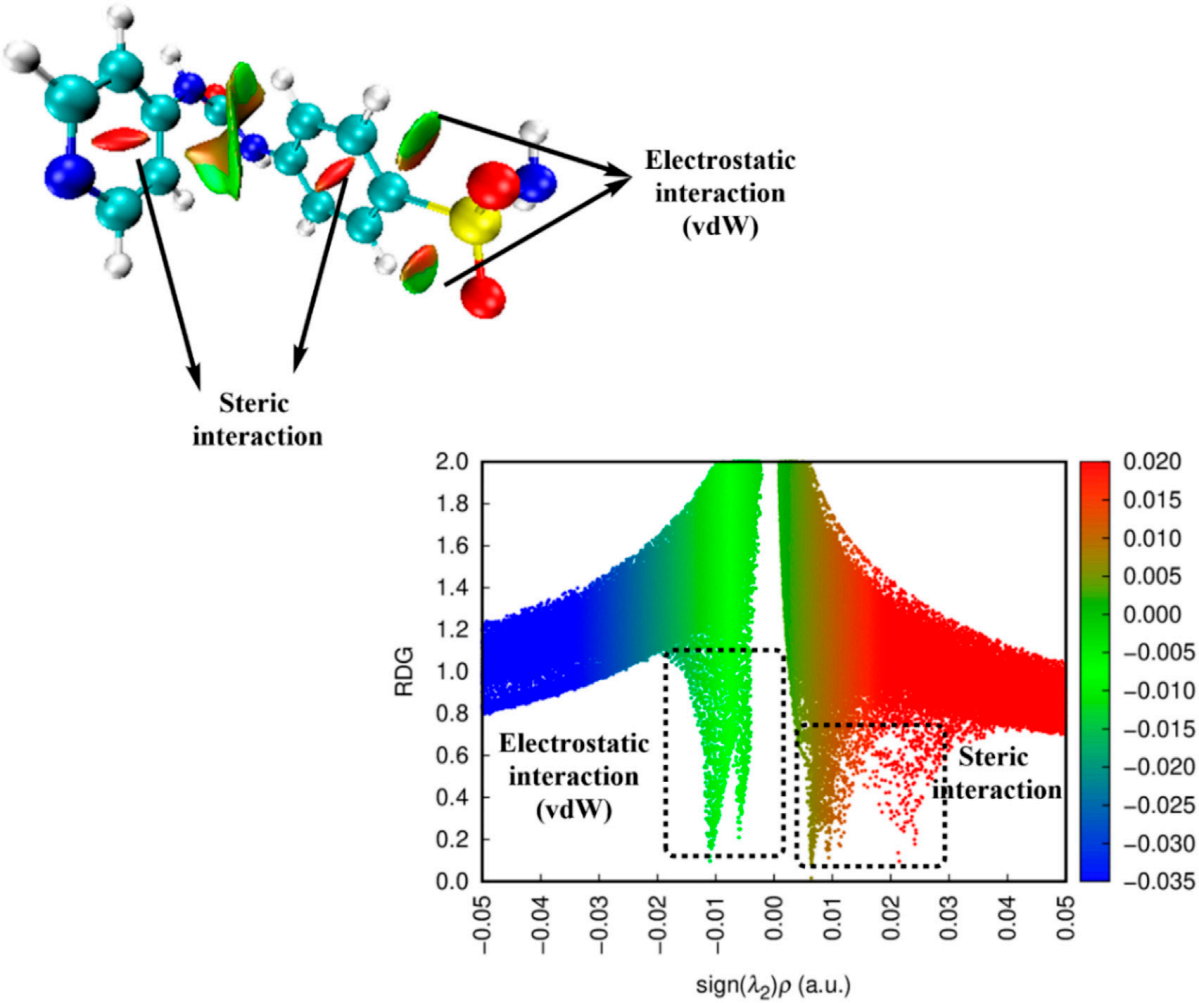
3D-NCI map and RDG plot of **Pyr**.

Utilizing reduced density gradient (RDG) research, noncovalent bond interactions (NCIs) between several molecular sites were identified. Different color codes were used to illustrate noncovalent interactions (El-Sayed et al., 2023). As illustrated in Figure 11, the interactions readily discernible on the surface of each molecule are vdW interactions with a green color scale and repulsion (steric) interactions with a red color scale. The sign (λ_2_)ρ, obtained by multiplying the electron density by the sign of the second Hessian eigenvalue, indicates the strength of the HB interaction in compounds. In this study, vdW is visible in the cage of C1-C2-S1 and C3-C2-S1. Other electrostatic interactions appear between the two phenyl rings. In this map, H-bond spikes do not appear in the chart; the high distance between H atoms and O atoms (around 2.4 Å) may lead to less sensitivity to be estimated with reduced density parameters. The unfavorable repulsion forces in the molecule were attributed to the steric effect of phenyl rings that export closed 6-atomic system constrain behavior. This type of steric interaction can be vanished by favorable electrostatic interactions.

#### 2.3.2 Molecular docking analysis

Molecular docking is a computational method used to predict the preferred orientation of a small molecule when bound to a receptor, such as a protein, and to evaluate the strength and stability of the interaction (Chang et al., 2023). For this study, the synthesized compound **Pyr** was docked into the active site of the carbonic anhydrase IX using PDB entry 5FL4, which contains the structure of CAIX bound to its cocrystallized ligand **9FK**. Autodock Vina, a widely used docking program, was employed to perform the docking simulations, and the results were visualized using Discovery Studio Visualizer, a tool that allows for detailed analysis of molecular interactions.

**FIGURE 12:**
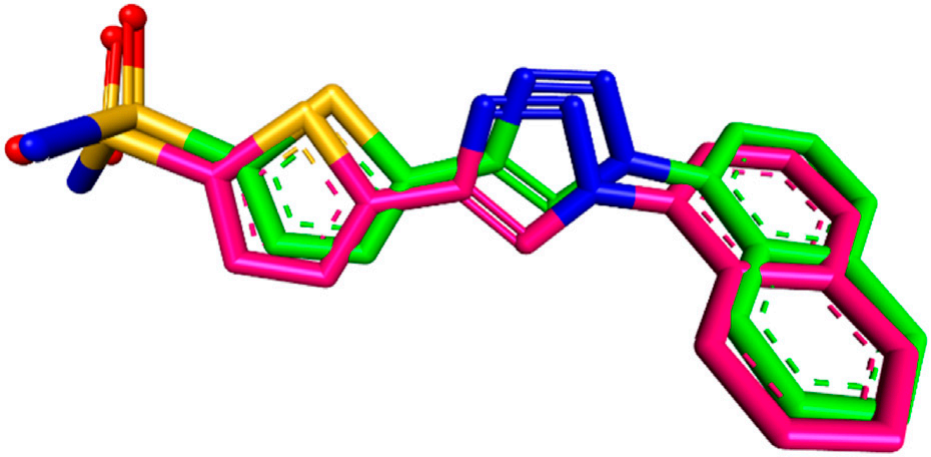
Superimposition of the redocked (green) and co-crystallized (magenta) poses of **9FK** inside the CAIX active site (RMSD = 0.9458 Å).

To ensure the accuracy of the docking procedure, the cocrystallized ligand **9FK** was redocked into the CAIX structure. The root-mean-square deviation (RMSD) between the original and redocked ligand poses was calculated to validate the results. An RMSD of 0.9458 Å, which is well below the typical threshold of 2 Å for reliable docking, indicated that the docking procedure and the employed parameters were accurate and capable of reproducing the experimental binding mode of the ligand. The binding affinity of the redocked ligand was found to be −8.6 kcal/mol, demonstrating a stable interaction with the CAIX enzyme. The superimposition of the redocked ligand with its original conformation is shown in Figure 12.

Following the validation of the docking protocol, **Pyr** was docked into the CAIX active site, and a comparative analysis was performed with acetazolamide, the reference compound used in the *in-vitro* CA inhibition assay. The binding affinity of **Pyr** was calculated to be −7.4 kcal/mol, indicating a moderately stable interaction with the CAIX enzyme, while acetazolamide showed a binding affinity of −7.7 kcal/mol, slightly higher than **Pyr**, which suggests a similar but slightly stronger binding interaction.

**FIGURE 13:**
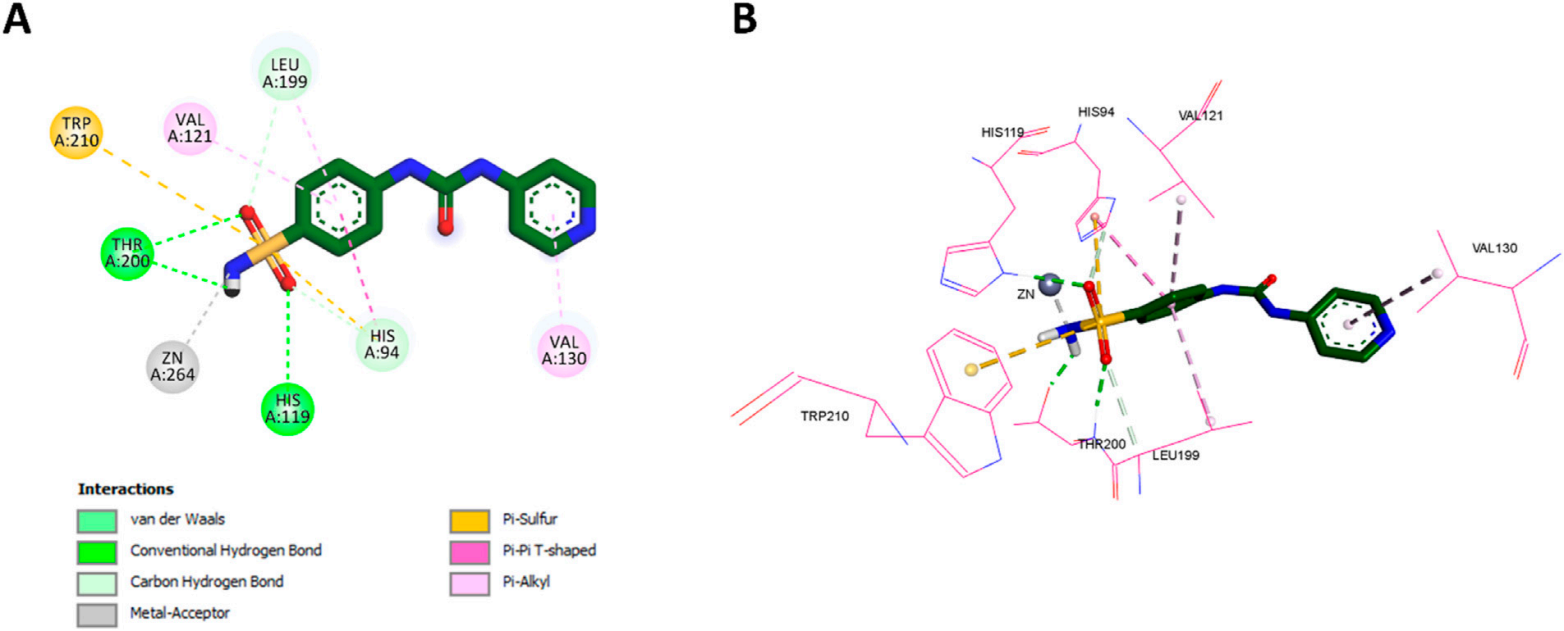
**(A)** 2D interaction diagram and **(B)** 3D binding mode of **Pyr** within the CAIX active site.

**Pyr** exhibited several key interactions that were similar to those of acetazolamide. Specifically, the NH2 group of **Pyr**’s sulfonamide interacted with the zinc ion in the active site, mirroring the interaction of acetazolamide, where the sulfonamide group serves as the zinc-binding moiety. Additionally, both the oxygen and NH2 groups of **Pyr**’s sulfonamide formed classical hydrogen bonds with THR200, as seen with acetazolamide. The sulfur atom of **Pyr**’s sulfonamide also engaged in Pi-sulfur interactions with Trp210 and His94, akin to acetazolamide’s interactions. The 2D and 3D interactions of **Pyr** are shown in Figure 13, illustrating these interactions in detail.

**FIGURE 14:**
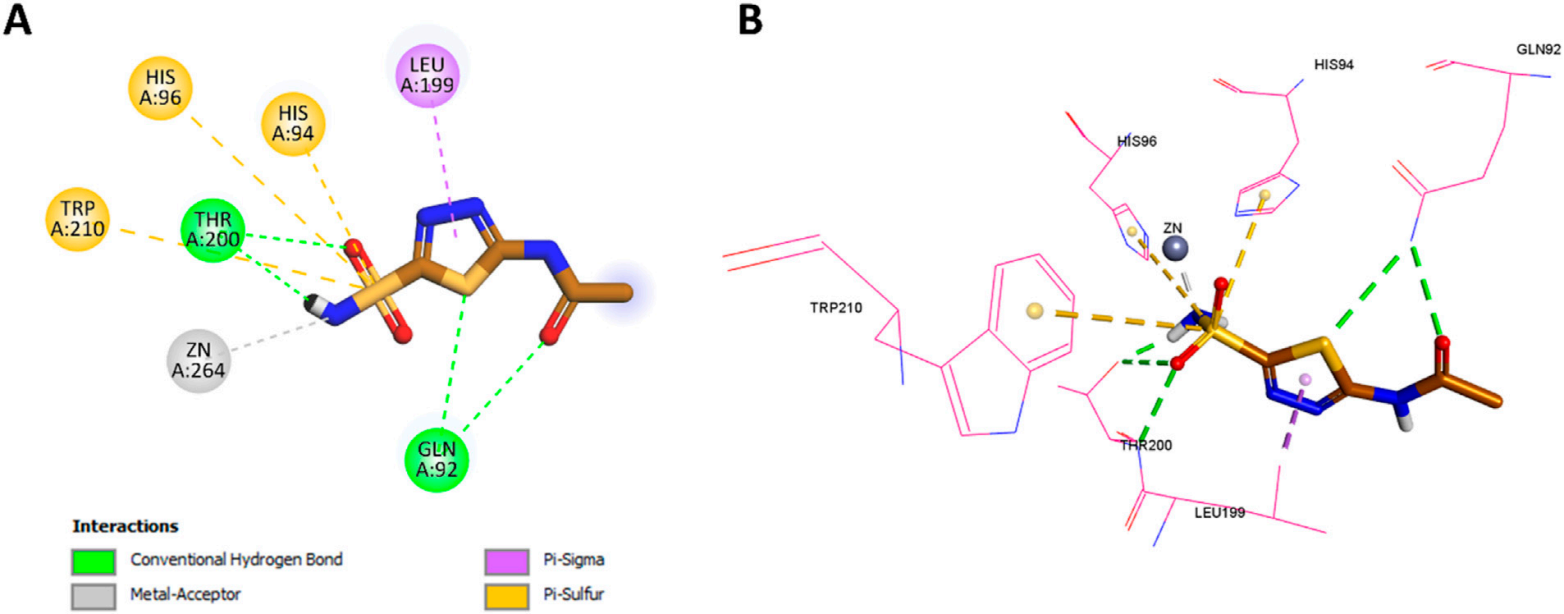
**(A)** 2D interaction diagram and **(B)** 3D binding mode of **Pyr** within the CAIX active site.

However, **Pyr** exhibited some unique interactions not observed with acetazolamide. The oxygen of **Pyr**’s sulfonamide formed a classical hydrogen bond with His119, a distinct interaction that was absent in acetazolamide. Moreover, **Pyr** formed non-classical hydrogen bonds with both Leu199 and His94, which were not seen in the acetazolamide docking. **Pyr**’s benzene ring demonstrated hydrophobic interactions with Leu199, a feature similar to the thiadiazole group of acetazolamide, but also formed additional hydrophobic interactions with both Val121 and His94, which were not present in acetazolamide. Finally, the pyridine ring (tail region) of **Pyr** exhibited Pi-alkyl (hydrophobic) interactions with Val130, a unique feature not observed in acetazolamide. The 2D and 3D interactions of acetazolamide are shown in Figure 14 for comparison.

#### 2.3.3 Drug likeness and ADMET predictions

**FIGURE 15:**
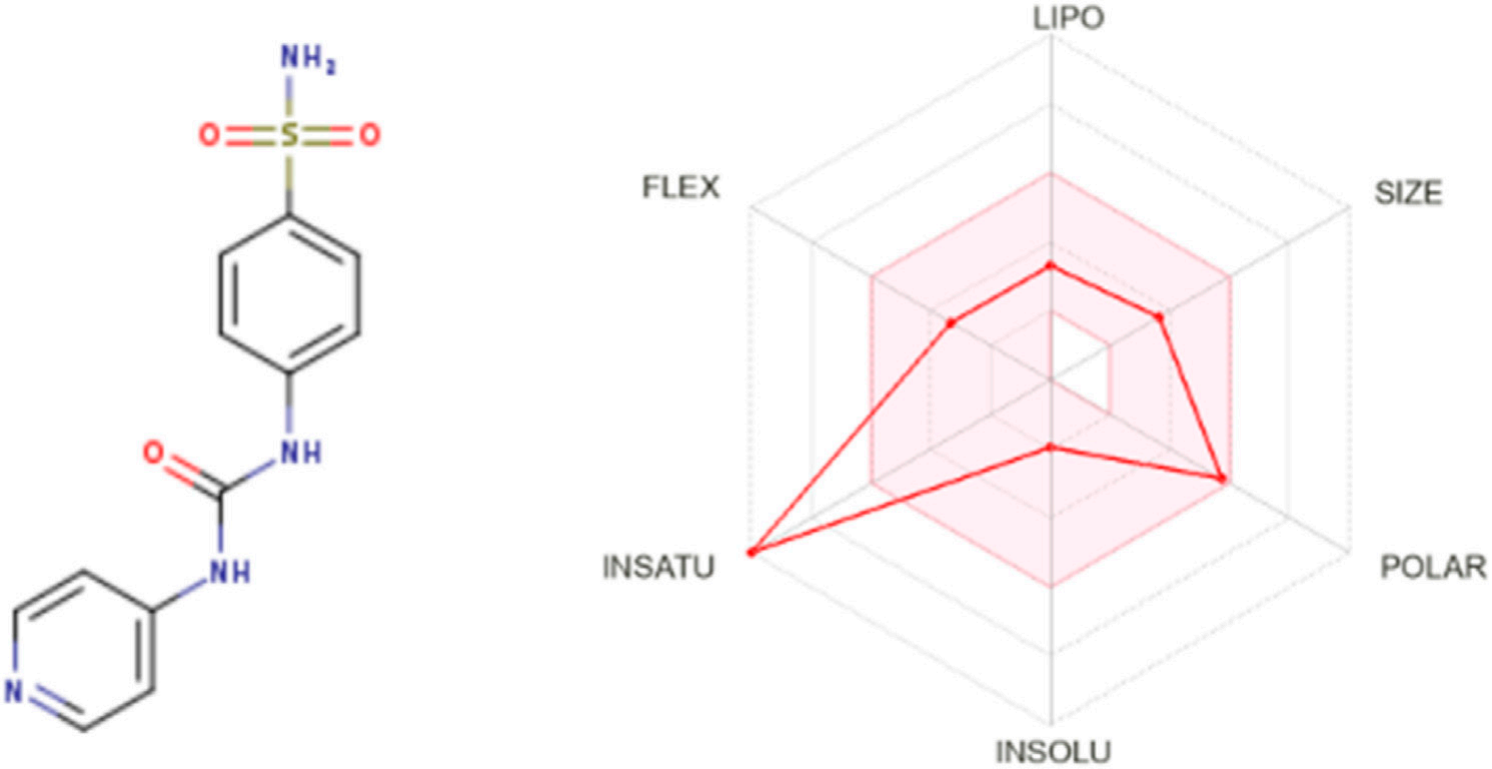
Bioavailability Radar model for **Pyr**.

**FIGURE 16:**
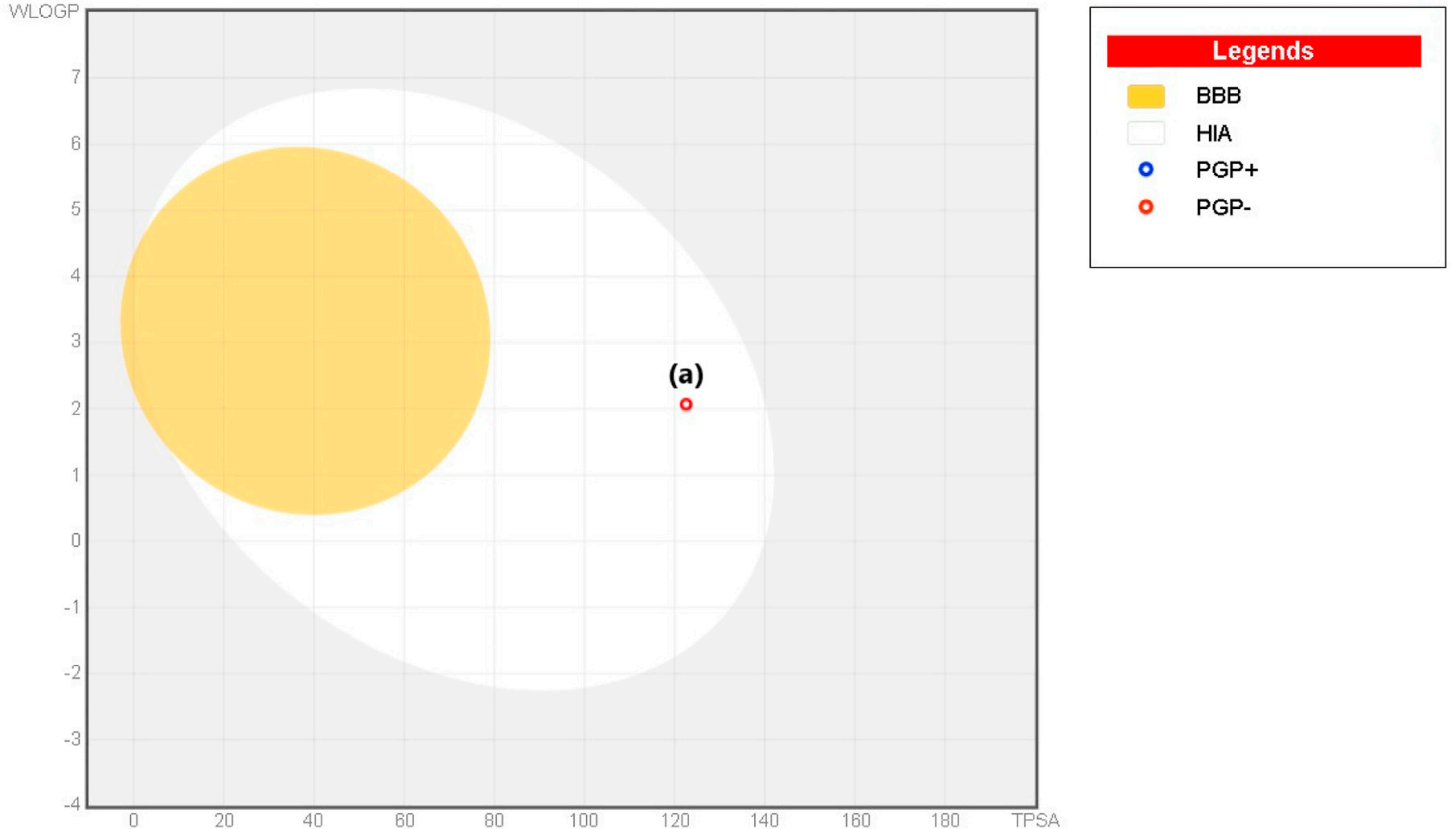
BOILED Egg plot of **Pyr**.

The Lipinski rule can assess the validity of the therapeutic properties in the compounds under investigation based on five determinants: molecular weight <500 Da, strong lipophilic qualities, such as LogP value <5, H-bond donors <5, H-bond acceptors <10 (Baell et al., 2013). The ligand under investigation may show signs of a drug when it meets more than two of these requirements. The drug-likeness features of PYR are shown in Table 7. It was calculated that the molecular weight of the ligand is 292.31 g/mol. It has five hydrogen bond acceptors and three hydrogen bond donors, where the investigated ligand meets these specifications. Excellent permeability across the cell membrane is indicated by the iLog P value of the molecule, which is determined to be within the range of less than 5, as predicted theoretically. It was found that the title ligand’s computed TPSA value was less than 140 Å^2^, which indicates good intestinal absorption values. Based on Lipinski’s rule, these findings support the hypothesis that PYR will absorb well. Significant bioavailability is predicted based on the TPSA and number of rotatable bond values. The primary focus of medical chemistry’s drug design, discovery, and contemporary drug development methods is on tiny molecules that resemble drugs and have high biological activity while being lowly harmful (Rautio et al., 2018). In silico prediction techniques, which enhance activity and toxicity study time and resource efficiency, can be used to identify (ADMET) traits (Ferreira and Andricopulo, 2019). The lipophilicity and pharmacokinetic parameters (ADMET features) are shown in Table 8. Drawing from the identified result, it can be shown that PYR exhibited a penetration capability that manifests as BBB permeant and GI absorption, indicating a reduced ability to cross the BBB and enter the central nervous system (Mani et al., 2023). The permeability value is −7.88 cm/s in human skin, showing that they cannot be absorbed within human skin. The propensity of a substance to inhibit five isoenzymes related to cytochrome P450, a major drug-metabolizing enzymes (CYP1A2, CYP2C19, CYP2C9, CYP2D6, and CYP3A4) can be evaluated using metabolic factors. CPY is utilized to develop medicinal effects and inhibit their capability. This trait is important for toxicity and other unfavorable medication interactions (Zhao et al., 2021). The predicted drug-potent ligand cannot affect these types of drug-metabolizing enzymes. **Pyr** exported a potential physicochemical characteristic that qualifies it for oral bioavailability. A suitable way to visualize this behavior is with the bioavailability radar, as shown in Figure 15. The pink zone in the bioavailability radar plot shows the optimal range for each of the six physicochemical parameters—size, solubility, lipophilicity, polarity, saturation, and flexibility. These characteristics are thought to be ideal for reaching the best oral bioavailability. A dependable model that accurately forecasts drug candidates’ absorption in the gastrointestinal system and their accessibility via the blood-brain barrier is the BOILED Egg method. **Pyr** was in a white zone, suggesting a high gastrointestinal absorption, as shown in Figure 16.

**TABLE 7:** Drug-likeness descriptors of the studied ligand **Pyr**.

Hydrogen bond acceptor (HBA)	5
Hydrogen bond donor (HBD)	3
Molecular Weight (MW)	292.31 g/mol
Topological polar surface area (TPSA)	122.56 Å^2^
No. of heavy atoms	20
No. of rotatable bonds	5
Partition coefficient, (ilog P)	0.73
Lipinski rule violations	yes
Bioavailability score	0.55

**TABLE 8:** Lipophilicity and pharmacokinetic parameters for **Pyr**.

XLOGP3	0.28
WLOGP	2.07
MLOGP	−0.09
Silicos-IT LogP	−0.62
Consensus LogP	0.47
CYP1A2 inhibitor	No
CYP 2C19 inhibitor	No
CYP 2C9 inhibitor	No
CYP 2D6 inhibitor	No
CYP 3A4 inhibitor	No
Skin permeability (logKp, cm/s)	−7.88
The blood-brain barrier (BBB+)	No
Human intestinal absorption (GI)	High

## 3 Experimental

### 3.1 Chemistry

All reactions were monitored using thin-layer chromatography (TLC) on Merck (Boston, MA, United States) 9385 pre-coated aluminum silica gel plates (Kieselgel 60) measuring 5 cm × 20 cm with a 0.2 mm layer thickness. Spots were visualized under UV light at a wavelength of 254 nm. Melting points were determined using Stuart’s electrothermal melting point apparatus and were uncorrected. Nuclear magnetic resonance (NMR) spectra for protons (^1H, 400 MHz) were recorded in DMSO-d6 on a Bruker AM400 spectrometer, with tetramethylsilane (TMS) as the internal standard. Chemical shift values are reported in parts per million (ppm), using DMSO-d6 as the solvent, and coupling constants (J) are expressed in hertz (Hz). Signal splitting patterns are described as follows: s (singlet), d (doublet), dd (doublet of doublets), t (triplet), q (quartet), m (multiplet), and brs (broad singlet). Intermediates **1-5** were synthesized according to the reported procedures in the literature (Maconi et al., 2002).

#### 3.1.1 Synthesis of 4-(3-(pyridin-4-yl)ureido)benzenesulfonamide (Pyr)

Freshly prepared azide derivative (**4**) (1.5 mmol, 0.178) was suspended in 10 mL of toluene, and the mixture was heated under reflux for 1 h until the evolution of N_2_ gas stopped. Then sulfanilamide (1 mmol, 0.172 g) was added portionwise to the solution, and the reaction mixture was heated under reflux for 2 h. The formed fluffy precipitate was filtered off and washed extensively with boiling toluene to remove unreacted isocyanate. The crude urea product was recrystallized from absolute ethanol.

White powder; 0.216 g, 74% yield; mp: 232°C–234°C; ^1^H NMR (400 MHz, DMSO-*d*6) δ = 9.10 (1H, s, urea-NH), 8.97 (1H, s, urea-NH), 8.34 (2H, d, pyridyl-2CH), 7.73 (2H, d, phenyl-2CH), 7.62 (2H, d, phenyl-2CH), 7.44 (2H, d, pyridyl-2CH), 7.24 (2H, s, pyridyl-NH_2_); ^13^C NMR (100 MHz, DMSO-*d*6) δ = 152.90, 150.40, 146.76, 143.78, 137.16, 126.92, 117.84, 112.15. Anal. Calcd for C_12_H_12_N_4_O_3_S (292.31): C, 49.31; H, 4.14; N, 19.17. Found: C, 49.43; H, 3.99; N, 19.40.

### 3.2 Biological evaluation

#### 3.2.1 Cell viability assay

The cell viability of colon HT29, breast MCF7, and prostate PC3 cancer cells, along with CCD-986sk normal cells, was evaluated for Pyr using MTT assay protocols as described in the literature (Ali et al., 2024). All cell lines were obtained from the Vacsera Cell Culture Library, Tissue Culture Unit, Cairo, Egypt, with ATCC certification. For further details, See Supplementary Appendix A in the supplementary data.

#### 3.2.2 Evaluation of carbonic anhydrase I, II, IV, and VII inhibition

According to the assay protocol, **Pyr** was incubated at room temperature for 10 min and analyzed in triplicate. Absorbance measurements were recorded at 405 nm in kinetic mode for 1 h at room temperature. The resulting data were plotted linearly to derive absorbance values, from which the IC_50_ value was determined using the slope of the plot (Abdelhakeem et al., 2024).

#### 3.2.3 Cell cycle analysis

The impact of Pyr on the cell cycle progression of PC3 cells was examined by analyzing DNA content with a flow cytometer, following the procedure outlined in the literature (Ali et al., 2024). For further details, See Supplementary Appendix A in the supplementary data.

#### 3.2.4 Determination of apoptosis

To detect apoptosis in PC3 cancer cells, the Annexin V FITC assay protocol was employed as described in the literature (Ali et al., 2024). For further details, See Supplementary Appendix A in the supplementary data.

#### 3.2.5 Effect of Pyr on caspase-3 activity and Bax and Bcl-2 protein expression levels

The impact of Pyr on caspase-3 protein levels, Bax, and Bcl-2 was evaluated following the protocols provided in the manufacturer’s kit instructions.

#### 3.2.6 Statistical analysis

Computerized Prism 5 program was used to statistically analyzed data using one-way ANOVA test followed by Tukey’s as post ANOVA for multiple comparison at P ≤ 0.05. Data were presented as mean ± SEM.

### 3.3 Molecular modeling studies

#### 3.3.1 DFT calculations

The target compound **Pyr** was subjected to a computational analysis using density functional theory (DFT) in order to provide fully optimized geometrical and electronic calculations using the hybrid B3LYP technique (Parr et al., 1980; Fayed et al., 2020). For further details, See Supplementary Appendix A in the supplementary data.

#### 3.3.2 Molecular docking

The docking procedure essentially follows a crucial path for extremely successful results. Consequently, the docking of the investigated complexes with control comparison was simulated using the AutoDock 4.2 program (Morris et al., 2009). Additionally, Discovery Studio was used to analyze and visualize docking data. For further details, See Supplementary Appendix A in the supplementary data.

#### 3.3.3 ADMET predictions

The ADMET parameters were calculated and *in silico* tests of the drug-like properties of **Pyr** were carried out using the open-source SwissADME server (Daina et al., 2017).

## 4 Conclusion

In conclusion, this study highlights the potential of a novel 4-pyridyl analog of SLC-0111 (Pyr) as a promising candidate for cancer therapy. **Pyr** exhibited selective cytotoxicity against various cancer cell lines (HT-29, MCF7, PC3) with reduced toxicity toward normal cells, demonstrating a favorable therapeutic index. Moreover, Pyr demonstrated potent and selective inhibition of tumor-associated carbonic anhydrase IX (CA IX) with an IC_50_ of 0.399 µg/mL with moderate inhibition of other CA isoforms I, II and XII. Further mechanistic investigations revealed its ability to induce G0/G1 phase cell cycle arrest and promote apoptosis in PC3 cells, supported by increased caspase-3 and caspase-9 activities and modulation of Bax/Bcl-2 and p53 protein levels. Molecular docking studies further validated Pyr’s strong binding affinity and selective inhibition of tumor-associated carbonic anhydrase IX. Additionally, ADMET predictions confirmed its drug-like properties and oral bioavailability. These findings establish **Pyr** as a viable candidate for further development in anticancer drug discovery and targeted therapy applications.

## Data Availability

The original contributions presented in the study are included in the article/Supplementary Material, further inquiries can be directed to the corresponding authors.

## Glossary

ADMET: Absorption, Distribution, Metabolism, Excretion, and Toxicity
AAZ: Acetazolamide
BBB: Blood-Brain Barrier
Bcl-2: B-cell lymphoma 2
CA: Carbonic Anhydrase
CA I: Carbonic Anhydrase Isoform I
CA II: Carbonic Anhydrase Isoform II
CA IX: Carbonic Anhydrase Isoform IX
CA XII: Carbonic Anhydrase Isoform XII
CAR-T: Chimeric antigen receptor T
CAIs: Carbonic Anhydrase Inhibitors
CCD-986sk: Human normal fibroblast cell line
DFT: Density Functional Theory
DMSO: Dimethyl Sulfoxide
ELF: Electron Localization Function
FMOs: Frontier Molecular Orbitals
GI: Gastrointestinal
HOMO: Highest Occupied Molecular Orbital
**IC**_**50**_: Half-maximal Inhibitory Concentration
Ki: Inhibition Constant
LUMO: Lowest Unoccupied Molecular Orbital
MCF7: Human breast cancer cell line
MEP: Molecular Electrostatic Potential
MTT: 3-(4,5-dimethylthiazol-2-yl)-2,5-diphenyltetrazolium bromide
NCI: Non-Covalent Interaction
NMR: Nuclear Magnetic Resonance
PC3: Human prostate cancer cell line
RMSD: Root-Mean-Square Deviation
TD-DFT: Time-Dependent Density Functional Theory
TLC: Thin-Layer Chromatography
TPSA: Topological Polar Surface Area
UV–Vis: Ultraviolet–Visible Spectroscopy
vdW: van der Waals
