# Supplementary material for: Discovery of a novel 4-pyridyl SLC-0111 analog targeting tumor-associated carbonic anhydrase isoform IX through tail-based design approach with potent anticancer activity
Source: Front Chem. 2025 Apr 4;13:1571646. doi: 10.3389/fchem.2025.1571646 (PMC12006758; doi:10.3389/fchem.2025.1571646)
Supplement: Supplementary file 1 [file DataSheet1.docx]

**Supporting information**

**Discovery of a novel 4-Pyridyl SLC-0111 Analog Targeting Tumor-Associated Carbonic Anhydrase Isoform IX Through Tail-Based Design Approach with Potent Anticancer Activity**

Hamada Hashem^1^, Shadwa Abdelfattah^2^, Hesham M. Hassan^3^, Ahmed Al-Emam^3^, Mohammed Alqarni^4^, Ghallab Alotaibi^5^, Ibrahim Taha Radwan^6^, Kirandeep Kaur^7^, Devendra Pratap Rao^*8^, Stefan Bräse^*9^, Abdullah Alkhammash^5^

^1^ Pharmaceutical Chemistry Department, Faculty of Pharmacy, Sohag University, Sohag 82524, Egypt

^2^ Department of Pharmaceutics and Industrial Pharmacy, Faculty of Pharmacy, Merit University (MUE), Sohag, 82755, Egypt

^3^ Department of Pathology, College of Medicine, King Khalid University, Asir 61421, Saudi Arabia

^4^ Department of Pharmaceutical chemistry, College of Pharmacy, Taif University, P.O. Box 11099, Taif 21944, Saudi Arabia

^5^ Pharmacology, College of Pharmacy, Al-Dawadmi Campus, Shaqra University, Shaqra, 11961, Saudi Arabia

^6^ Supplementary General Sciences Department, Faculty of Oral and Dental Medicine, Future University in Egypt, Cairo 11835, Egypt

^7^ Department of Chemistry, Maharaja Ranjit Singh Punjab Technical University, Bathinda 151001, Punjab, India

^8^Coordination Chemistry Laboratory, Department of Chemistry, Dayanand Anglo-Vedic (PG) College, Kanpur 208001, Uttar Pradesh, India

^9^ Institute of Biological and Chemical Systems, Functional Molecular Systems (IBCS-FMS), Karlsruhe Institute of Technology (KIT), Kaiserstrasse 12, 76131 Karlsruhe, Germany

*To whom correspondence should be addressed

**Appendix A**

**1. Chemistry**

**
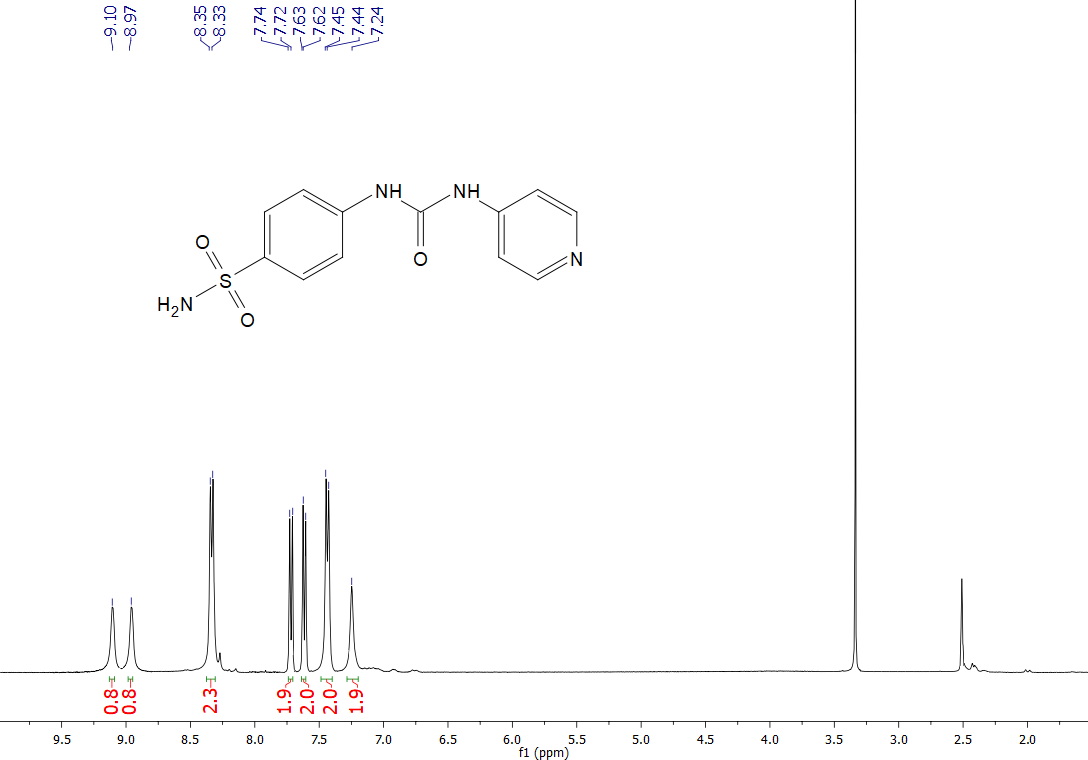
**

**Figure S1. ^1^H-NMR spectrum of the target compound** (400 MHz, DMSO-*d*_6_)


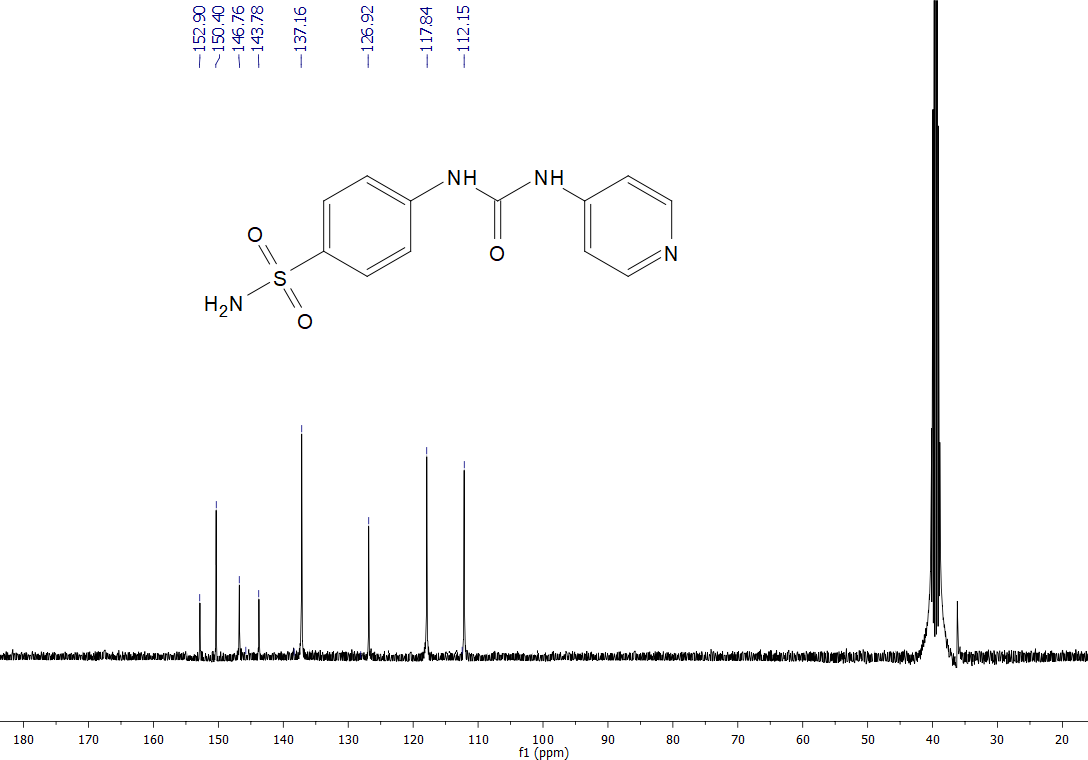


Figure S2. ^13^ C-NMR spectrum of the target compound (100 MHz, DMSO-*d*_6_)

**2. Biology**

**2.1. Cell Viability Assay**

The IC_50_ values of the target compound were determined for colon HT29, breast MCF7, and prostate PC3 cancer cells, as well as CCD-986sk normal cells, using established MTT assay protocols. All cell lines were obtained from the Vacsera Cell Culture Library, Tissue Culture Unit, Cairo, Egypt, with ATCC certification. The IC50 values, representing the concentration that inhibits 50% of cell growth, were calculated based on the mean of three independent experiments.

Cells were cultured in DMEM (Invitrogen/Life Technologies) supplemented with 10% FBS (Hyclone), 10 mg/ml insulin (Sigma), and 1% penicillin-streptomycin. Cells were seeded at a density of 1.2–1.8 × 10,000 cells per well in 96-well plates with 100 µL of complete growth medium and 100 µL of the test compound. After 24 hours of incubation, 100 µL of serially diluted sterile test compounds were added to achieve final concentrations ranging from 0.01 to 100 µM, with growth media serving as the negative control. Following another 24-hour incubation, supernatants were removed, and cells were prepared for MTT testing.

HT29, MCF7, PC3, CCD-986sk and cells were trypsinized and washed with calcium/magnesium-free PBS (pH 7.2). Experiments were conducted in a laminar flow hood, using cells in the logarithmic growth phase to ensure cell counts did not exceed 10⁶ cells/cm². Each experiment included a blank control with complete media but no cells. MTT [M-5655] was reconstituted with 3 mL of medium or balanced salt solution lacking phenol red and serum. Reconstituted MTT was added to the culture medium at 10% of its volume, and cultures were incubated for 2 to 4 hours, depending on cell type and density.

After incubation, formazan crystals were dissolved by adding an equal volume of MTT Solubilization Solution [M-8910] to the original culture medium. Absorbance was measured spectrophotometrically at 570 nm, with background absorbance at 690 nm subtracted. Data from all experiments were recorded, and cell viability percentages were calculated.

**2.2. Evaluation of Carbonic anhydrase I, II, IV, and VII inhibition**

According to the assay protocol, **Pyr** was incubated at room temperature for 10 minutes and analyzed in triplicate. Absorbance measurements were recorded at 405 nm in kinetic mode for 1 hour at room temperature. The resulting data were plotted linearly to derive absorbance values, from which the IC_50_ value was determined using the slope of the plot.

**2.3. Cell cycle analysis**

PC3 cells were treated with Pyr at its IC_50_ concentration for 24 hours. After treatment, the cells were washed twice with ice-cold phosphate-buffered saline (PBS, pH 7.4, Sigma-Aldrich, Inc.), collected by centrifugation, and fixed in 66% ice-cold ethanol. The cells were subsequently washed with PBS, resuspended in a solution containing 0.1 mg/mL RNase for 30 minutes, stained with 50 µg/mL propidium iodide (PI), and analyzed using the FACSCalibur flow cytometry system (Becton Dickinson, Franklin Lakes, NJ, USA). Cell cycle distributions were determined with CellQuest software (Becton Dickinson).

**2.4. Determination of apoptosis**

The Annexin V-FITC Apoptosis Detection Kit (Catalog #: K101-25, Bio Vision Research Products, Mountain View, CA, USA) operates on the principle that during the early stages of apoptosis, phosphatidylserine (PS) translocates from the inner leaflet of the plasma membrane to the extracellular surface. This relocation allows PS to be detected by Annexin V, a protein with high affinity for PS, when conjugated with a fluorescent compound. The staining process is quick, taking only 10 minutes, and apoptosis detection is performed using flow cytometry. The kit differentiates between apoptotic and necrotic cells based on Annexin V-FITC and propidium iodide (PI) labeling. Early apoptotic cells are Annexin V-positive and PI-negative, while late apoptotic cells are both Annexin V-positive and PI-positive.

For the experiment, cells (5 × 10⁵) were cultured in triplicate in Dulbecco’s Modified Eagle Medium (DMEM) at 37 °C in a 5% CO₂ atmosphere for 24 hours to allow for attachment. Afterward, the medium was replaced with DMEM containing the test compound at its IC50 concentration, and the cells were incubated for 48 hours. Following incubation, cells were harvested, washed with cold PBS, and resuspended in binding buffer. Annexin V and PI were added, and the cells were incubated at 4 °C in the dark for 30 minutes for staining. Flow cytometric analysis was performed using the FACS Calibur system (Becton Dickinson, Franklin Lakes, NJ, USA) on a minimum of 10⁴ cells. Dot plots were generated, and the total fraction of apoptotic cells was quantified as described.

**3. Molecular Modeling**

**3.1. DFT Calculations**

The synthesized heterocyclic compound was subjected to a computational analysis using density functional theory (DFT) in order to provide fully optimized geometrical and electronic calculations using the hybrid B3LYP technique. The basis set 6-311++G (d,p) was performed using Gaussian 09 software, and MEP analysis was performed in order to assess the significant nucleophilic and electrophilic sites for the optimized structures. The best structure of the heterocyclic compound and its energy excitation levels were shown using Chemcraft and VMD software to study electronic behavior. The 6-311++G(d,p) harmonic vibrational modes were calibrated or corrected for vibrational data using the 0.967 multiplying vibrational scale factor (<https://cccbdb.nist.gov/vibscalejust.asp>)**.** Using Multiwfn software, more topological studies on reduced density gradient/non-covalent interaction (RDG/NCI) were conducted to illustrate intramolecular interaction types and electron localization function (ELF) to enhance the comprehension of interaction types and bond nature between atoms in the investigated heterocyclic system.

**3.2. Molecular Docking and ADMET calculations**

The docking procedure essentially follows a crucial path for extremely successful results. Consequently, the docking of the investigated complexes with control comparison was simulated using the AutoDock 4.2 program. Additionally, Discovery Studio (https://www.3ds.com/products-services/biovia/) was used to analyze and visualize docking data. Carbonyic anhydrase isoform IX (ID: 5FL4) was selected based on the experimental results to perform the in-silico studies. The Protein Data Bank website provided a noteworthy comparison of the protein of interest. The target protein was first prepared by eliminating water molecules and any unwanted atoms, before the optimized compounds were docked, under specific circumstances. Following the addition of polar H-atoms, the protein charge was modified, and the complexes were applied as pdbqt extension files. The projected active locations were located, and the size of the grid box were established. The grid box size was estimated, where the dimensions of the target enzyme are 40 × 40 × 40 Å, with a 0.375 Å spacing, and its grid centers xyz-coordinates were -27.429, 12.486, -26.988. The binding affinity mode was previously believed to be the Genetic Algorithm (LGA)**.**

In the process of developing novel drugs, Lipinski's rule of five is very useful for figuring out the bioavailability of bulk materials. The ADMET parameters were calculated and in silico tests of the drug-like properties of the target compound were carried out using the open-source SwissADME server**.**
